# HIV-1 mRNA Knockdown with CRISPR/Cas9 Enhances Neurocognitive Function

**DOI:** 10.21203/rs.3.rs-3266933/v1

**Published:** 2023-10-16

**Authors:** Kristen A McLaurin, Hailong Li, Kamel Khalili, Charles F. Mactutus, Rosemarie M. Booze

**Affiliations:** University of South Carolina; University of South Carolina; Lewis Katz School of Medicine at Temple University; University of South Carolina; University of South Carolina

**Keywords:** Cell Culture, In Situ Hybridization, Gene Editing, Microglia, Prepulse Inhibition

## Abstract

Mixed glia are infiltrated with HIV-1 virus early in the course of infection leading to the development of a persistent viral reservoir in the central nervous system. Modification of the HIV-1 genome using gene editing techniques, including CRISPR/Cas9, has shown great promise towards eliminating HIV-1 viral reservoirs; whether these techniques are capable of removing HIV-1 viral proteins from mixed glia, however, has not been systematically evaluated. Herein, the efficacy of adeno-associated virus 9 (AAV9)-CRISPR/Cas9 gene editing for eliminating HIV-1 mRNA from cortical mixed glia was evaluated *in vitro* and *in vivo*. *In vitro*, a within-subjects experimental design was utilized to treat mixed glia isolated from neonatal HIV-1 transgenic (Tg) rats with varying doses (0, 0.9, 1.8, 2.7, 3.6, 4.5, or 5.4 μL) of CRISPR/Cas9 for 72 hours. Dose-dependent decreases in the number of HIV-1 mRNA, quantified using an innovative *in situ* hybridization technique, were observed in a subset (i.e., *n*=5 out of 8) of primary mixed glia. *In vivo*, HIV-1 Tg rats were retro-orbitally inoculated with CRISPR/Cas9 for two weeks, whereby treatment resulted in profound excision (i.e., approximately 53.2%) of HIV-1 mRNA from the mPFC. Given incomplete excision of the HIV-1 viral genome, the clinical relevance of HIV-1 mRNA knockdown for eliminating neurocognitive impairments was evaluated via examination of temporal processing, a putative neurobehavioral mechanism underlying HIV-1 associated neurocognitive disorders (HAND). Indeed, treatment with CRISPR/Cas9 partially restored the developmental trajectory of temporal processing. Proof-of-concept studies, therefore, support the susceptibility of mixed glia to gene editing and the potential of CRISPR/Cas9 to serve as a novel therapeutic strategy for HAND, even in the absence of full viral eradication.

## INTRODUCTION

The current treatment regimen for HIV-1 seropositive individuals includes a cocktail of two to four drugs, collectively known as combination antiretroviral therapy (cART), which act to suppress viral replication (e.g., Gulick et al., 1997). cART revolutionized the treatment of HIV-1 and transformed the disease into a chronic, manageable condition, whereby HIV-1 seropositive individuals have life expectancies similar to those of seronegative persons (Marcus et al., 2020). Nevertheless, disease management is challenged by poor adherence to cART, which is associated with viral rebound (e.g., Maina et al., 2020). Furthermore, despite treatment with cART, HIV-1 seropositive individuals are disproportionately afflicted by comorbidities (Marcus et al., 2020; e.g., Liver Disorders: Morales et al., 2022; Cardiovascular Disease: Alonso et al., 2019, Touloumi et al., 2020) and exhibit high rates of neurocognitive impairments (e.g., Wang et al., 2020). In light of these observations, scientists have been on a quest to develop a functional cure (i.e., long-term HIV-1 control in the absence of cART) for HIV-1.

Elimination of HIV-1 viral reservoirs, which are established early in the course of infection (Gantner et al., 2023) and persist despite cART (e.g., Chun et al., 1997; Finzi et al., 1997; Wong et al., 1997), presents a key challenge to the development of a functional cure. A viral reservoir, as defined by Eisele and Siliciano (2012), includes infected cell populations that allow long-term persistence (i.e., years) of replication-competent HIV-1 in HIV-1 seropositive individuals on cART. Although the primary HIV-1 viral reservoir resides in memory CD4^+^ T cells, there is compelling evidence for the existence of additional viral reservoir sites, including the central nervous system (CNS; for review, Churchill et al., 2016, Wallet et al., 2019). Microglia, in particular, are the resident immune cells of the CNS whose lifespan is approximately 4.2 years (Réu et al., 2017). Further, the migration of HIV-1-infected monocytes across the blood-brain barrier (for review, Williams et al., 2012) results in the infection of microglia evidenced by the presence of replication-competent HIV-1 (i.e., HIV-1 DNA) in both untreated (Thompson et al., 2011) and virally suppressed (Ko et al., 2019) HIV-1 seropositive individuals. The ability of microglia to harbor HIV-1 DNA, in conjunction with their long lifespan, meets the criteria for a viral reservoir based on the definition established by Eisele and Siliciano (2012). Eradication of HIV-1 from the CNS, therefore, is likely to be of fundamental import for the successful implementation of a functional cure strategy.

Three broad strategies have been pursued to completely eradicate the HIV-1 genome, including stem cell transplantation (Hutter et al., 2009; Gupta et al., 2019; Jensen et al., 2023; Hsu et al., 2023), “shock and kill” approaches (e.g., for review, Ait-Ammar et al., 2020), and gene editing (e.g., Hu et al., 2014; Qu et al., 2013). Successful eradication of the HIV-1 genome has been achieved in four individuals, whereby three individuals received a CCR5Δ32 homozygous allogeneic adult stem cell transplant (Hutter et al., 2009; Gupta et al., 2019; Jensen et al., 2023) and one adult was cured via a CCR5Δ32/Δ32 haplo-cord transplant (Hsu et al., 2023). Despite the recent success of a combined haploidentical and cord blood transplant, which has the potential to increase donor cell availability, widespread implementation of this approach remains impractical. The “shock and kill” approach utilizes latency-reversing agents (LRA; e.g., Tat-R5M4: Geng et al., 2016) to reactivate HIV-1 transcription, thereby enabling the immune system to kill latently infected cells. Although treatment with LRAs induces reactivation of viral transcription in latently infected cells, the viral reservoir size is not reduced in a clinically meaningful manner (e.g., Rasmussen et al., 2014; Gruell et al., 2022). Further, the CNS presents an additional obstacle for the “shock and kill” approach, as it may induce unintended adverse effects, including harmful neuroinflammation (Gama et al., 2017). Programmable nuclease-based gene editing utilizing zinc finger (Qu et al., 2013) or clustered, regularly-interspaced, short palindromic repeats (CRISPR)/ CRISPR-associated 9 (Cas9) nucleases (e.g., Hu et al., 2014; Kaminski et al., 2016a; Dash et al., 2019), however, represent a promising strategy to eradicate the HIV-1 genome from infected cells.

CRISPRs, which were first identified in the genome of *Escherichia coli* (Ishino et al., 1987), are a family of repetitive deoxyribonucleic acid (DNA) sequences characterized by direct repeats interspaced by similarly sized non-repetitive elements clustered in at least one loci on the chromosome. Upstream of the CRISPR array are CRISPR-associated (*cas*) genes that encode proteins with nuclease and helicase domains (e.g., Cas9: Makarova et al., 2002), The Cas9 protein, in particular, contains an HNH and RuvC-like nuclease domain; distinct active sites that are utilized to cleave double-stranded DNA (dsDNA; Jinek et al., 2012; Gasiunas et al., 2012). Fundamentally, co-expression of Cas9 and custom-engineered guide ribonucleic acid (gRNA) molecule(s) induces highly efficient site-specific alterations in the target DNA affording a novel tool for gene editing (Jinek et al., 2012; Cong et al., 2013; Jinek et al., 2013; Mali et al., 2013).

Indeed, Cas9-mediated genome editing affords a novel strategy to excise the integrated HIV-1 proviral genome *in vitro* and *in vivo* (for review, Bhowmik & Chaubey, 2022). The development of single gRNA molecules to target the flanking HIV-1 long terminal repeat (LTR) sequences provided a fundamental proof-of-concept, whereby treatment cleaved viral DNA *in vitro* and inhibited viral gene expression (Ebina et al., 2013; Hu et al., 2014; Kaminski et al., 2016a; Yin et al., 2016). The potential for InDel generation, and thus an “escape mutant”, with a single gRNA (Wang G et al., 2016; Wang Z et al., 2016), however, necessitates a multiplex gRNA configuration; a configuration that also efficaciously eradicates the integrated HIV-1 proviral genome *in vitro* (e.g., Hu et al., 2014; Kaminski et al., 2016a; Yin et al., 2016). Fundamentally, delivery of the Cas9/gRNA system using an adeno-associated virus (AAV) vector excises the HIV-1 provirus *in vivo* (Kaminski et al., 2016b; Yin et al., 2017; Dash et al., 2019). The efficacy of Cas9/gRNAs for the excision of constitutively expressed HIV-1 viral proteins from the CNS, however, remains understudied.

Thus, complementary *in vitro* and *in vivo* aims were undertaken to address this fundamental knowledge gap using the HIV-1 transgenic (Tg) rat. The HIV-1 Tg rat, originally reported by Reid et al. (2001), expresses HIV-1 viral proteins constitutively throughout development (Peng et al., 2010; Abbondanzo & Chang, 2014), whereby HIV-1 mRNA is predominantly expressed in microglia in a restricted, region-specific manner (Li et al., 2021). First, the utility of the Cas9/gRNA system to excise HIV-1 viral proteins *in vitro* from primary mixed glia was established using a dose-response experimental paradigm. Second, the efficacy of the Cas9/gRNA system for HIV-1 mRNA excision *in vivo* was evaluated. Third, the clinical relevance of HIV-1 mRNA knockdown for mitigating neurocognitive impairments was examined using a longitudinal evaluation of temporal processing; temporal processing is a potential neurobehavioral mechanism underlying higher-order cognitive processes (McLaurin et al., 2019a). Establishing the susceptibility of HIV-1-infected mixed glia to gene editing via AAV9-CRISPR/Cas9 is fundamental to the widespread implementation and success of the therapeutic approach.

## MATERIALS AND METHODS

### Experimental Design

**Figure 1:**
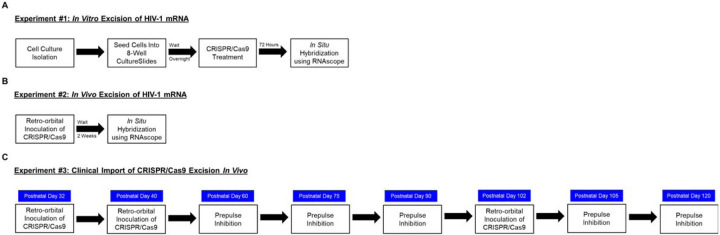
Experimental Design Schematic.

A schematic of the experimental design for the complementary *in vitro* and *in vivo* aims is illustrated in Fig. 1.

### Animals

For Experiment #1: *In Vitro* Excision of HIV-1 mRNA, breeding of Fischer F344/N HIV-1 Tg pups was conducted at the University of South Carolina, whereby a Fischer F344/N control female (Envigo Laboratories Inc., Indianapolis, IN, USA) was paired with an HIV-1 Tg male. Primary mixed glia were isolated from no more than one male and one female HIV-1 Tg rat from each litter to preclude violation of the assumption of independence.

For Experiment #2: *In Vivo* Excision of HIV-1 mRNA and Experiment #3: Clinical Import of CRISPR/Cas9 Excision *In Vivo* male and female HIV-1 Tg and control (Fischer F344/N) animals were procured from Envigo Laboratories during early adolescence. Animals were pair-housed with animals of the same sex for the duration of the study.

During both experiments, HIV-1 Tg and control rats had *ad libitum* access to rodent food (Pro-Lab Rat, Mouse, Hamster Chow #3000) and water. Animals were housed in AAALAC-accredited facilities with environmental conditions targeted at 21°C ± 2°C, 50% ± 10% relative humidity, and a 12-h light:12-h dark cycle with lights on at 700 h (EST). Guidelines established by the National Institutes of Health in the Guide for the Care and Use of Laboratory Animals were utilized for the maintenance of all animals. The University of South Carolina Institutional Animal Care and Use Committee approved the project protocol under Federal Assurance (#D16–00028).

#### Experiment #1: In Vitro Excision of HIV-1 mRNA

##### Primary Mixed Glial Cell Cultures

The prefrontal cortex was dissected from male (*n* = 4) and female (*n* = 4) neonatal (i.e., Postnatal Day 1–3) HIV-1 Tg rat pups for the isolation of mixed glia. Following dissection, brain tissue was transferred into a 15 mL centrifuge tube with Hank’s balanced salt solution (HBSS) buffered with 10 mM HEPES (GIBCO Life Technologies, Grand Island, NY, USA) and 0.25% Trypsin/EDTA (GIBCO Life Technologies); the centrifuge tube was incubated at 37°C in 5% CO_2_/95% room air-humidified incubator for 20 minutes. Tissue was dissociated using trituration and transferred into a 75 mm poly-L-lysine coated flask with DMEM/F12 medium supplemented with 10% fetal bovine serum (FBS). DMEM/F12 medium supplemented with 10% FBS was changed the day after isolation and supplemented every three to seven days. Upon reaching at least 90% confluency, mixed glia were reseeded into poly-L-lysine coated 8-well CultureSlides (Corning, Corning, NY, USA) for experimentation. Cultures were maintained in the incubator (i.e., 37°C in 5% CO_2_ and 95% humidity) at all times.

##### CRISPR/Cas9 Plasmid and Treatment

A detailed description of the guide RNA (gRNA) design, construction of the CRISPR/Cas9 expression plasmid, and adeno-associated virus 9 (AAV_9_) vector have been previously published (Hu et al., 2014). In brief, gRNAs with high guide efficiency (i.e., to maximize on-target activities in the HIV-1 genome) and specificity (i.e., to minimize off-target effects in the human genome) were selected based on screening using the Broad Institute gRNA designer tool; screening which yielded two gRNAs targeting the HIV-1 LTR promotor region and the *gag* gene. A plasmid carrying the SaCas9 endo-nuclease gene under the CMV promoter and gRNAs was packaged into an AAV_9_ serotype (Vigene Biosciences Inc., Milton Park, Abingdon, UK). An AAV_9_ delivery vector was selected for the delivery of CRISPR/Cas9 given its robust transduction efficiency into the central nervous system (Foust et al., 2009). Cortical mixed glial cell cultures were treated for 72 hours with one of seven doses of AAV_9_-CRISPR/Cas9 (i.e., 0, 0.9, 1.8, 2.7, 3.6, 4.5, or 5.4 μL).

##### RNAscope In Situ Hybridization

A highly sensitive and specific RNAscope *in situ* hybridization technique (Advanced Cell Diagnostics, Inc., Newark, CA, USA) was utilized to detect the presence of HIV-1 mRNA. Seventy-two hours after CRISPR/Cas9 treatment, 8-well CultureSlides were submerged into phosphate-buffered saline (PBS; 1x) and cells were fixed using 10% neutral buffered formalin (Sigma-Aldrich, St. Louis, MO, USA). After washing with PBS, cells were dehydrated using an increasing ethanol (EtOH) gradient (i.e., 50% EtOH for 5 minutes, 70% EtOH for 5 minutes, 100% EtOH for 5 minutes, 100% EtOH for 10 minutes). Slides were stored at −20°C until further experimentation.

Upon continuation of the *in situ* hybridization assay, cells were rehydrated using a decreasing EtOH gradient (i.e., 70% EtOH for 2 minutes, 50% EtOH for 2 minutes) followed by PBS (10 minutes). A hydrophobic barrier was created around each well using the ImmEdge Hydrophobic Barrier PAP Pen (Vector Laboratories, Burlingame, CA, USA) and allowed to dry completely. After being rinsed with PBS, the RNAscope Protease III reagent (Advanced Cell Diagnostics, Inc.) was added to cells in each well and incubated in the HybEZ Humidity Control Tray (Advanced Cell Diagnostics, Inc.). Slides were washed twice by agitating them in PBS.

RNA *in situ* hybridization was subsequently conducted using the RNAscope Multiplex Fluorescent Assay (Advanced Cell Diagnostics, Inc., Newark, CA, USA). Detailed methodological procedures for the RNAscope Multiplex Fluorescent Assay are presented in Li et al. (2018) with minor modifications. Specifically, cells were hybridized with a specific probe for HIV-1 mRNA (i.e., Probe-V-HIV1-CladeB-vif-vpr-tat-rev-vpu-env-nef-tar, Catalogue Number: 444061, Advanced Cell Diagnostics, Inc.). Additionally, following all amplification steps, cells were counterstained with DAPI for 30 seconds prior to mounting (ProLong Gold Antifade, Invitrogen, Carlsbad, CA, USA) and coverslipping.

##### HIV-1 mRNA Quantification

Multiple z-stack images were obtained from high confluency areas, as evidenced by DAPI staining, in each well using a Nikon TE-200E confocal microscope system controlled by Version 3.81b of Nikon’s EZ-C1 software. Images were captured at 60x using an oil objective with a numerical aperture of 1.4 and z-plane intervals of 0.15 μm. Fluorophore excitation of the HIV-1 mRNA probe and DAPI staining were accomplished using an argon helium neon laser (Emission: 488 nm) and HeNe helium-neon (Emission: 633 nm), respectively.

HIV-1 mRNA exhibited a “discrete dots” (Li et al., 2018) staining pattern affording an opportunity to quantify the number of HIV-1 mRNA. Z-stack images were blinded to prevent experimental bias. The number of HIV-1 mRNA signals were counted from two z-stack images per well based on established selection criteria (i.e., high cell confluency, bright HIV-1 mRNA signal). To preclude violation of the assumption of independence, the number of HIV-1 mRNA signals from the two z-stack images per well was averaged (Denenberg, 1984; Wears, 2002).

#### Experiment #2: In Vivo Excision of HIV-1 mRNA

##### Retro-orbital CRISPR/Cas9 Inoculation

Adult male and female HIV-1 Tg animals were randomly assigned to receive retro-orbital injections of either CRISPR/Cas9 (i.e., AAV9-SaCas9-sgRNA-HIV-LTR1/GagD, 4.7 × 10^12^ gc/ml; *n* = 4; Male: *n* = 2, Female: *n* = 4) or saline (*n* = 4; Male: *n* = 2, Female: *n* = 4). After inhalant anesthesia was induced with sevoflurane, the animal was placed laterally with the injection-eye facing upwards. A 1cc tuberculin syringe with a 26G needle was slowly inserted into the medial canthus of the eye at a 45° angle to the nose. 50 μL of CRISPR/Cas9 or saline was gently injected into the retro-orbital vessels. Animals were allowed to recover in a heat-regulated warm chamber.

##### Tissue Preparation

Approximately two weeks after CRISPR/Cas9 inoculation, anesthesia was induced using 5% sevoflurane, and HIV-1 Tg rodents were humanely sacrificed. Within five minutes of sacrifice, the brain was harvested from the skull and frozen in liquid nitrogen for 15-sec. The brain was sliced into coronal sections using a cryostat (30 μm) and transferred onto slides (SuperFrost Plus Slides, Thermo Fisher Scientific, Hampton, NH, USA) for *in situ* hybridization.

##### In Situ Hybridization using RNAscope

HIV-1 mRNA expression in the mPFC was evaluated using a highly specific and sensitive RNA *in situ* hybridization technique. Minor modifications to the RNA *in situ* hybridization technique, described in detail by Li et al. (2018), were implemented in the present study. Specifically, the coronal sections including the mPFC (approximately 3.7 mm to 2.2 mm anterior to Bregma; Paxinos & Watson, 2014) were hybridized with a probe specific for HIV-1 mRNA (i.e., Probe-V-HIV1-CladeB-vif-vpr-tat-rev-vpu-env-nef-tar, Catalogue Number: 444061, Advanced Cell Diagnostics, Inc.).

#### Experiment #3: Clinical Import of CRISPR/Cas9 Excision In Vivo

##### Retro-orbital Injections of CRISPR/Cas9

Upon arrival at the animal vivarium, F344/N control and HIV-1 Tg animals were randomly assigned to receive retro-orbital injections of CRISPR/Cas9 (HIV-1 Tg, *n* = 8; Male: *n* = 4, Female: *n* = 4), the viral vector HM4d (Control, *n* = 8; Male: *n* = 4, Female: *n* = 4), or saline (Control, *n* = 8; Male: *n* = 4, Female: *n* = 4; HIV-1 Tg, *n* = 8; Male: *n* = 4, Female: *n* = 4). Animals were inoculated at postnatal day (PD) 32, 40, and 102. Retro-orbital inoculations were conducted as described in Experiment #2.

##### Assessments of Temporal Processing

###### Apparatus

The startle platform (SR-Lab Startle Reflex System, San Diego Instruments, Inc., San Diego, CA) and Plexiglas test cylinder were enclosed within a double-walled isolation cabinet (External Dimensions: 81 × 81 × 116 cm; Industrial Acoustic Company, Inc., Bronx, NY) that provided 30 dB(A) of sound attenuation. In the absence of any stimuli, the ambient sound level within the isolation cabinet was 22 dB(A). Auditory prepulse and startle stimuli were presented using a high-frequency loudspeaker (Model #40–1278B, Radio Shack, Fort Worth, TX) that was mounted 30 cm above the Plexiglas test cylinder. A 22 lux white LED light, which was mounted on the wall of the isolation chamber, was utilized to present visual prepulse stimuli. A deflection of the Plexiglas test cylinder was produced by the animal’s response to the auditory startle stimulus. Response signals were converted into analog signals by a piezoelectric accelerometer integral to the Plexiglas test cylinder, digitized (12-bit A to D), and saved to a hard disk.

###### Prepulse Inhibition of the Auditory Startle Response

Prior to an assessment of temporal processing, animals were habituated using the test procedure previously described (McLaurin et al., 2017a). Temporal processing was subsequently evaluated using a 30-minute cross-modal prepulse inhibition experimental paradigm (described in detail by McLaurin et al., 2017a). In brief, after a 5-minute acclimation period, the startling stimulus was presented for six trials; trials which were separated by a fixed 10-sec intertrial interval. During the subsequent 72 testing trials, a visual or auditory prepulse was presented prior to the auditory startling stimulus at interstimulus intervals (ISIs) of 0, 30, 50, 100, 200, or 4000 msec; the 0 and 4000 msec ISI trials served as control trials to provide a reference auditory startle response (ASR) within the test session. Counterbalancing (i.e., ABBA) was implemented for the presentation of auditory and visual prepulse trials, while a Latin-square experimental design was utilized for the presentation of ISIs within 6-trial blocks. The intertrial interval for testing trials was variable, whereby it ranged from 15 to 25 sec. Mean peak ASR amplitude values were collected for subsequent analyses.

### Statistical Analysis

Statistical analyses, including analysis of variance (ANOVA) and regression techniques, were conducted using SPSS Statistics 28 (IBM Corporation, Somer, NY) and GraphPad Prism 5 (La Jolla, CA), respectively. GraphPad Prism 5 was also used for the creation of figures. An α criterion of *p* ≤ 0.05 was set for the establishment of statistical significance.

Two approaches were utilized to evaluate the susceptibility of mixed glia to gene editing via CRISPR/Cas9 *in vitro*. First, statistical analyses were conducted on all primary mixed glia (*n* = 8). Second, when the overall analysis failed to reveal any statistically significant effects, complementary analyses that included a subset of animals were conducted. Indeed, the number of HIV-1 mRNA from primary mixed glia cultured from a subset of neonatal HIV-1 Tg rats was examined dependent upon which phenomena (i.e., excision at high (5.4 μL; *n* = 3) or low (1.8 μL; *n* = 2) dose of CRISPR/Cas9) they exhibited. Data were analyzed using a repeated measures ANOVA, whereby CRISPR/Cas9 dose served as a within-subjects factor. Two CRISPR/Cas9 doses were missing from primary mixed glia cultured from one neonatal HIV-1 Tg rat exhibiting significant excision when treated with 5.4 μL of CRISPR/Cas9; mean imputation was utilized for the repeated-measures ANOVA. Complementary linear regression analyses were also conducted.

The *in vivo* efficacy of CRISPR/Cas9 was evaluated using a univariate ANOVA, whereby the number of HIV-1 mRNA in the mPFC served as the dependent variable of interest. Treatment served as the between-subjects factor.

Further, the clinical relevance of HIV-1 mRNA knockdown for mitigating neurocognitive impairments was evaluated using linear regression analyses, whereby two dependent variables of interest (i.e., Startle Response, PPI) were derived from the ISI function (McLaurin et al., 2019b) and examined using *a priori* planned comparisons. Specifically, startle response was calculated as an average between the 0 and 4000 msec ISI trials, whereas the area of inflection of the ASR amplitude response curve was calculated as an index of PPI (McLaurin et al., 2016). Furthermore, the genotype deficit (i.e., Control vs. HIV-1 Tg Saline) and effect of CRISPR/Cas9 (i.e., Control vs. HIV-1 Tg CRISPR/Cas9) were established using *a priori* planned comparisons. Given that the viral vector HM4d had no statistically significant effect on either of the dependent variables of interest, the control data presented and analyzed were collapsed across viral infusion.

## RESULTS

### Experiment #1: In Vitro Excision of HIV-1 mRNA

#### CRISPR/Cas9 efficaciously excised HIV-1 viral proteins from primary mixed glia in a subset of neonatal HIV-1 Tg rats.

**Figure 2:**
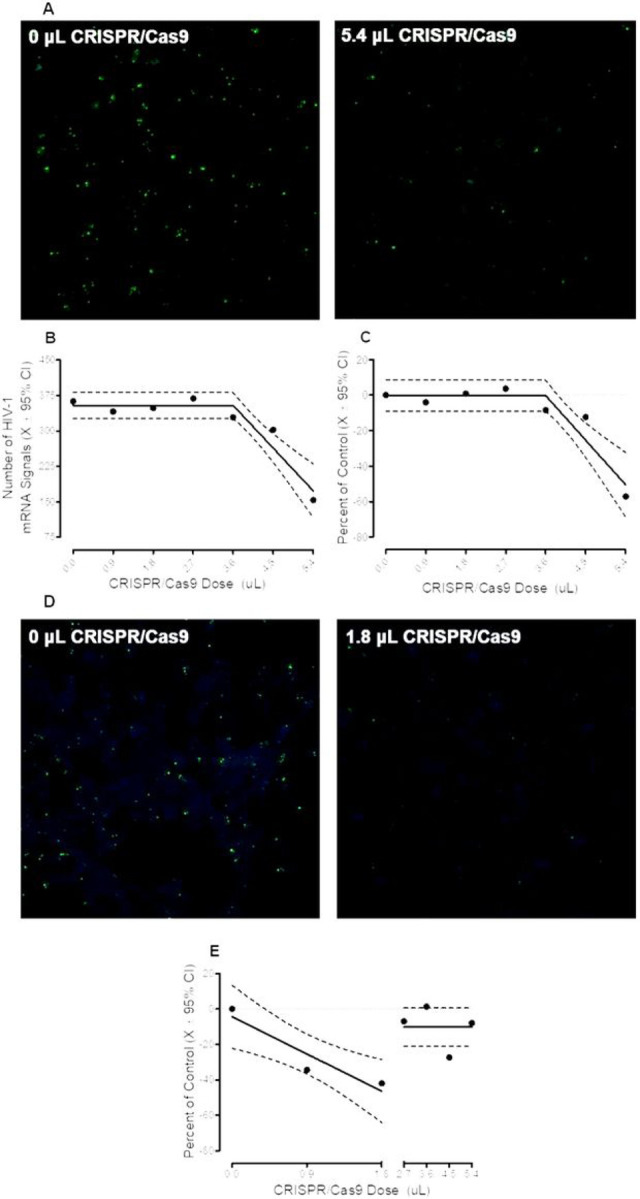
HIV-1 mRNA was efficaciously excised from primary mixed glia in a subset (i.e., *n*=5 out of 8) of neonatal HIV-1 Tg rats, whereby two prominent phenomena were observed. **(A)** First, pronounced excision of HIV-1 mRNA was observed in mixed glia cultured from three neonatal HIV-1 Tg rat pups following treatment with 5.4 μL of CRISPR/Cas9. **(B-C)** Quantification of the number of HIV-1 mRNA signals revealed dose-dependent excision efficient that was well-described by a segmental linear regression; observations which were confirmed by expressing the data as a percentage change relative to the respective untreated control. **(D)** Second, low doses of CRISPR/Cas9 (i.e., 1.8 μL) were sufficient to excise mixed glia cultured from two neonatal HIV-1 Tg rat pups. **(E)** Expression of the data as a percentage change relative to the respective untreated control revealed a linear decrease from 0 to 1.8 μL.

Primary mixed glia were cultured from the prefrontal cortex of HIV-1 Tg rats, which express seven of the nine HIV-1 viral proteins constitutively throughout development (Peng et al., 2010; Abbondanzo & Chang, 2014). Cells were treated with one of seven doses of CRISPR/Cas9 (i.e., 0, 0.9, 1.8, 2.7, 3.6, 4.5, or 5.4 μL) for 72 hours, after which time the number of HIV-1 mRNA, visualized using RNAscope *in situ* hybridization, were quantified. Pronounced HIV-1 mRNA expression was observed in cells not treated with CRISPR/Cas9 (i.e., 0 μL; Number of HIV-1 mRNA (Mean ± Standard Error of the Mean): 375.4 ± 40.5) A subset (i.e., *n* = 5 out of 8) of primary mixed glia isolated from neonatal HIV-1 Tg rats exhibited dose-dependent decreases in HIV-1 mRNA following CRISPR/Cas9 treatment (Fig. 2). Indeed, two phenomena were observed, whereby HIV-1 mRNA was efficaciously excised following treatment with either high (i.e., 5.4 μL; *n* = 3) or low (i.e., 1.8 μL; *n* = 2) doses of CRISPR/Cas9.

First, in mixed glia cultured from three neonatal HIV-1 Tg rat pups, pronounced excision of HIV-1 mRNA was observed following treatment with 5.4 μL of CRISPR/Cas9 (Fig. 2A). The number of HIV-1 mRNA signals was well-described by a segmental linear regression (*R*^2^ ≥ 0.90; Fig. 2B), whereby a linear decrease in the number of HIV-1 mRNA signals was observed from 3.6 μL to 5.4 μL (Main Effect of Dose with a Primary Quadratic Component: *F*(1,2) = 182.5, *p* ≤ 0.005, η_p_^2^ = 0.989). Expressing the data as a percentage change relative to the respective untreated control confirmed dose-dependent excision efficiency that was best-fit using a segmental linear regression (*R*^2^ ≥ 0.88; Fig. 2C). Indeed, at the 5.4 μL dose of CRISPR/Cas9, the average excision efficiency of HIV-1 mRNA from primary mixed glia was 57.1% (Range: 46.9–70.5%).

Second, HIV-1 mRNA was efficaciously excised from mixed glia cultured from two neonatal HIV-1 Tg rat pups after treatment with low (i.e., 1.8 μL) doses of CRISPR/Cas9 (Fig. 2D). From 0 μL to 1.8 μL, the number of HIV-1 mRNA signals, expressed as a percentage change relative to the respective untreated control, decreased in a linear manner (Best-Fit Function: First-Order Polynomial, *R*^2^ ≥ 0.81; Fig. 2E). The average excision efficiency of HIV-1 mRNA from primary mixed glia at the 1.8 μL dose of CRISPR/Cas9 was 41.9% (Range: 39.7–44.2%).

### Experiment #2: In Vivo Excision of HIV-1 mRNA

#### Retro-orbital inoculation with CRISPR/Cas9 significantly decreases HIV-1 mRNA in the medial prefrontal cortex of HIV-1 Tg rats.

**Figure 3:**
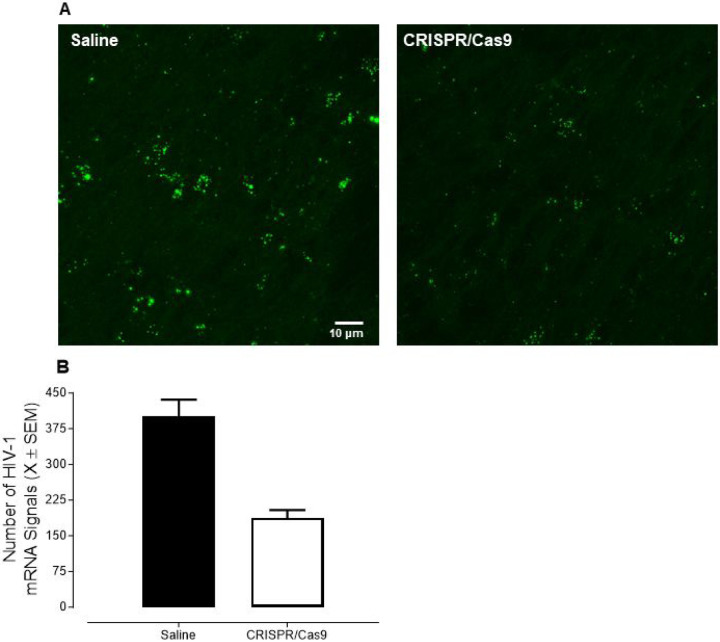
Efficacious excision of HIV-1 mRNA from the central nervous system of HIV-1 transgenic rats was observed *in vivo*. **(A)** Prominent HIV-1 mRNA expression was observed in HIV-1 Tg animals treated with saline; expression levels which were significantly decreased by retro-orbital inoculation with CRISPR/Cas9. **(B)** Quantification of the number of HIV-1 mRNA signals confirms significant excision following CRISPR/Cas9 treatment, whereby excision efficiency was approximately 53.2%.

Subsequent *in vivo* experiments were undertaken to further evaluate the ability of CRISPR/Cas9 to efficaciously excise HIV-1 mRNA from the CNS of HIV-1 Tg rats (Saline: *n* = 4, Male: *n* = 2, Female: *n* = 2; CRISPR/Cas9: *n* = 4, Male: *n* = 2, Female: *n* = 2). CRISPR/Cas9 or saline was retro-orbitally inoculated into the orbital venous plexus, which flows through the superior ophthalmic vein to the cavernous sinus (i.e., part of the brain’s dural venous sinuses; Ngnitewe Massa et al., 2022). Approximately two weeks after retro-orbital inoculation with CRISPR/Cas9 or saline, *in situ* hybridization techniques were utilized to visualize and quantify the number of HIV-1 mRNA. Profound HIV-1 mRNA expression was observed in HIV-1 Tg animals treated with saline (Fig. 3A). HIV-1 mRNA expression was significantly decreased by treatment with CRISPR/Cas9 (Main Effect of Treatment: *F*(1,6) = 29.3, *p* ≤ 0.002, η_p_^2^ = 0.830), whereby excision efficiency was approximately 53.2%. Taken together, these proof-of-concept observations *in vitro* and *in vivo* support the susceptibility of mixed glia to efficacious gene editing via AAV9-CRISPR/Cas9.

### Experiment #3: Clinical Import of CRISPR/Cas9 Excision In Vivo

#### Complete excision of the HIV-1 genome may be unnecessary to enhance neurocognitive function.

Given incomplete excision, subsequent *in vivo* studies were conducted to establish the clinical relevance of HIV-1 mRNA knockdown. For example, the mitigation of HIV-1-associated neurocognitive disorders (HAND), which afflict approximately 50% of HIV-1 seropositive individuals (Wang et al., 2020), has the potential to improve the quality of life for HIV-1 seropositive individuals. Therefore, following retro-orbital inoculation with CRISPR/Cas9, the viral vector HM4d, or saline, temporal processing, a potential neurobehavioral mechanism underlying HAND (McLaurin et al., 2019a), was evaluated longitudinally using PPI. Two dependent variables of interest (i.e., Startle Response and Prepulse Inhibition) were examined using *a priori* planned comparisons to establish an HIV-1 genotype effect (i.e., Control vs. HIV-1 Tg Saline) and the magnitude of the treatment effect (i.e., Control vs. HIV-1 CRISPR/Cas9). Given that the viral vector HM4d had no statistically significant effect on either of the dependent variables of interest, the control data presented and analyzed were collapsed across viral infusion.

**Figure 4:**
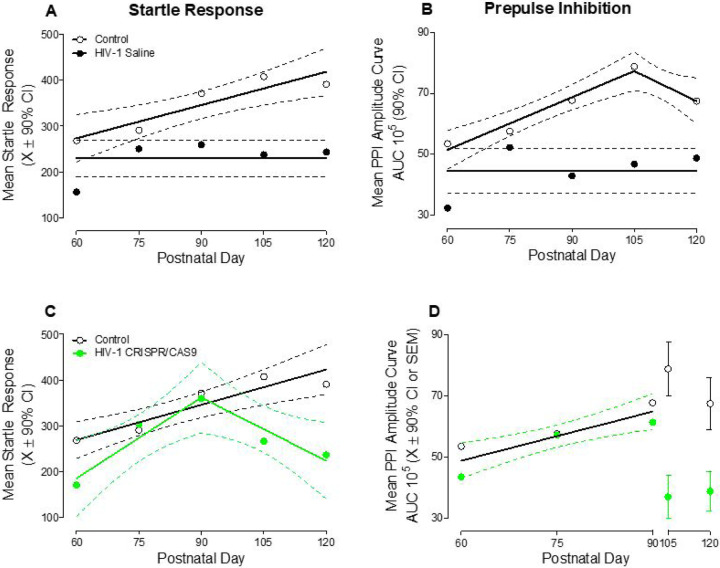
Even in the absence of complete excision of the HIV-1 viral genome, treatment with CRISPR/Cas9 is clinically relevant. Startle response **(A, C)** and prepulse inhibition **(**PPI; **B, D)** were derived from the interstimulus interval function. HIV-1 Tg animals treated with saline exhibited profound deficits in the development of both startle response **(A)** and PPI **(B)**. Treatment with CRISPR/Cas9, however, partially restored the developmental trajectory of startle response **(C)** and PPI **(D)**.

Constitutive expression of the HIV-1 transgene induces pronounced alterations in the development of temporal processing, indexed using startle response (Fig. 4A) and PPI (Fig. 4B). HIV-1 Tg animals treated with saline failed to exhibit any statistically significant development of either startle response or PPI from PD 60 to PD 120, whereby data were well-described by a horizontal line. In sharp contrast, the development of mean startle response and PPI in control animals was well-described by a first-order polynomial (*R*^2^ ≥ 0.84) and segmental linear regression (*R*^2^ ≥ 0.96), respectively. Regression analyses, therefore, illustrate the prominent temporal processing deficit induced by HIV-1 viral protein exposure.

Retro-orbital inoculation with CRISPR/Cas9, however, partially restored the development of temporal processing in HIV-1 Tg animals, evidenced by measures of startle response (Fig. 4C) and PPI (Fig. 4D). From PD 60 to PD 90, the developmental trajectory of startle response and PPI in HIV-1 Tg animals treated with CRISPR/Cas9 resembles that of control animals. Indeed, HIV-1 Tg animals inoculated with CRISPR/Cas9 exhibited a linear increase in startle response through PD 90 followed by a subsequent decrease (Best Fit Function: Segmental Linear Regression, *R*^2^ ≥ 0.90). Overlapping 90% confidence intervals between HIV-1 Tg animals treated with CRISPR/Cas9 and control animals support statistically indistinguishable developmental trajectories. With regards to PPI, the linear increase from PD 60 to PD 90 observed in both HIV-1 Tg animals treated with CRISPR/Cas9 and control animals was well-described by a global fit (i.e., First Order Polynomial: *R*^2^ ≥ 0.78), whereby no statistically significant differences in the parameters of the function were observed (*p* ≥ 0.05). Taken together, even in the absence of complete excision, the knockdown of HIV-1 mRNA enhances neurocognitive function for a period of time.

## DISCUSSION

Proof-of-concept studies support the susceptibility of mixed glia to gene editing via CRISPR/Cas9, whereby pronounced, albeit incomplete, excision of the HIV-1 viral genome was observed both *in vitro* and *in vivo*. *In vitro*, a subset (i.e., *n* = 5 out of 8) of primary mixed glia isolated from neonatal HIV-1 Tg rats exhibited dose-dependent decreases in the number of HIV-1 mRNA following CRISPR/Cas9 treatment. Efficacious excision, defined as at least a 35% decrease in the number of HIV-1 mRNA, from primary mixed glia occurred at either the 1.8 μL or 5.4 μL dose. *In vivo*, retro-orbital inoculation of CRISPR/Cas9 into the orbital venous plexus of HIV-1 Tg rats resulted in profound excision (i.e., approximately 53.2%) of HIV-1 mRNA from the mPFC. Nevertheless, excision of the complete HIV-1 viral genome may be unnecessary for the mitigation of HAND, as the developmental trajectory of temporal processing was partially restored in HIV-1 Tg animals treated with CRISPR/Cas9. Thus, independent of full viral eradication, gene editing via CRISPR/Cas9 may afford a novel therapeutic strategy for HAND.

Significant individual variability in the efficacy of CRISPR/Cas9 in primary mixed glial cultures (i.e., pronounced excision in only a subset (*n* = 5 out of 8 cultures) may be due, at least in part, to the dsDNA repair pathway utilized by cells. Efficacious gene editing using CRISPR/Cas9 enzymes relies upon the cleavage of dsDNA at locations precisely defined by custom-engineered gRNA molecule(s); genome integrity and the viability of the cell, however, are dependent upon proper repair of dsDNA breaks. dsDNA breaks are primarily repaired via one of three processes, including non-homologous end joining (NHEJ), microhomology-mediated end joining (MMEJ), or homology-directed repair (HDR). Although NHEJ and MMEJ are recognized as the main repair mechanisms for dsDNA breaks, these pathways, which act by re-ligating broken DNA ends in the absence of a DNA template from an exogenous donor, are error-prone (for review, Xue & Greene, 2021). Indeed, with specific regard to HIV-1, mutations induced by NHEJ processes facilitated viral escape (Wang G et al., 2016). In sharp contrast, HDR is the preferred repair mechanism for CRISPR/Cas9-induced dsDNA breaks, as it more accurately repairs the genome via a homologous donor template; upregulation of end joining dsDNA repair mechanisms (i.e., NHEJ, MMEJ) in eukaryotic cells, however, limits the extent to which HDR is utilized (Xue & Greene, 2021). Hence, regulating the pathway used to repair dsDNA breaks during genome editing has the potential to enhance the efficacy and precision of CRISPR/Cas9 genome editing.

The utilization of primary mixed glial cultures, rather than purified microglia, to evaluate the efficacy of CRISPR/Cas9 is of fundamental importance. Under homeostatic conditions *in vivo*, the morphology of microglial cells is characterized by a small cell body and very fine, ramified processes. Purified microglia in culture, however, display an amoeboid-like phenotype (Cristóvão et al., 2010, Caldeira et al., 2014) and express a lysosomal enzyme (i.e., CD68) associated with phagocytic activity during the first ten days *in vitro* (Cristóvão et al., 2010). Changes to environmental tissue conditions (i.e., *in vitro* vs. *in vivo*) also induces pronounced alterations in the transcriptomes and epigenomic features of microglia (Bohlen et al., 2017; Gosselin et al., 2017), whereby gene expression alterations occur in a time-dependent manner (Bohlen et al., 2017). Indeed, serum supplementation, which has historically been utilized to stimulate cellular growth (Puck et al., 1958) and considered fundamental to the viability of cells in culture (Puck et al., 1958), is involved in the perturbation of microglial properties *in vitro* (Bohlen et al., 2017; Montilla et al., 2020). In light of these findings, serum-free conditions resembling the physiological condition of cerebrospinal fluid have been defined; conditions that also promote microglial survival and a ramified microglial morphology *in vitro* (Bohlen et al., 2017; Montilla et al., 2020). Nevertheless, results derived from only primary microglial cultures should be interpreted with caution, as *in vitro* models are not without limitation.

Similarly, the validity of *in vivo* studies is dependent upon the utilization of a biological system that recapitulates key aspects of the clinical phenotype. Characteristics of rats, including their effective regulation of *tat* transactivation and the presence of compatible cellular cofactors that permit the production of HIV-1 viral proteins (Yedavalli et al., 2003), renders them an appropriate species for a transgenic HIV-1 biological system. In 2001, Reid et al. (2001) reported the development of a hemizygous HIV-1 Tg rat that contains a non-infectious transgenic construct consisting of a *gag-pol* deleted (i.e., non-replicative) HIV-1 provirus regulated by the human viral long terminal repeat. In total, seven of the nine HIV-1 genes (i.e., *env, nef, rev, tat, vif, vpr, and vpu*) are expressed constitutively throughout development in the HIV-1 Tg rat (Peng et al., 2010 Abbondanzo & Chang, 2014) resembling HIV-1 seropositive individuals on cART. Features of the HIV-1 Tg rat support its utility as a biological system to model neuroHIV (for review, Vigorito et al., 2015) and noninfectious comorbidities (Denaro et al., 2020). Indeed, many of the neuropathological hallmarks of neuroHIV are recapitulated in the HIV-1 Tg rat, including the expression of HIV-1 mRNA in microglia (Clinical: Ko et al., 2019, Li et al., 2023; HIV-1 Tg Rat: Li et al., 2021), profound synaptodendritic injury (Clinical: Moore et al., 2006, Weiss et al., 2021; HIV-1 Tg Rat: Roscoe et al., 2014, McLaurin et al., 2018a, McLaurin et al., 2021), and progressive neurocognitive impairments (Clinical: Heaton et al., 2015, Sacktor et al., 2016, Rubin et al., 2017, Gott et al., 2017; HIV-1 Tg Rat: McLaurin et al., 2016; McLaurin et al., 2019c). Hence, the HIV-1 Tg rat afforded an *in vivo* biological system with compelling face validity to further evaluate the susceptibility of mixed glia to gene editing via CRISPR/Cas9.

HIV-1 Tg rats retro-orbitally inoculated with CRISPR/Cas9 exhibited a pronounced decrease in the number of HIV-1 mRNA in the mPFC relative to their saline-inoculated counterparts. Recombinant adeno-associated viral (rAAV) vectors, which are utilized to deliver exogenous DNA to rodents (e.g., CRISPR/Cas9), can be intravenously delivered via the tail vein (Foust et al., 2009; Gray et al., 2011), facial vein (Foust et al., 2009), or retro-orbital venous sinus (Prabhakar et al., 2019; for protocol, Prabhakar et al., 2021; Present Study). During retro-orbital inoculation, rAAV vectors are slowly injected into the orbital venous plexus, which flows through the superior ophthalmic vein to the cavernous sinus (i.e., part of the brain’s dural venous sinuses; Ngnitewe Massa et al., 2022) providing direct access to the CNS. The advantages of retro-orbital, rather than tail vein, injections cannot be understated, as retro-orbital injections are suitable for all ages (e.g., Newborn: Gruntman et al., 2017, Adults: for protocol, Yardeni et al., 2011) and induce a lower stress response (Steel et al., 2008). Indeed, retro-orbital inoculation of HIV-1 Tg rats with CRISPR/Cas9 illustrates the utility of the delivery approach, as well as the susceptibility of mixed glia to gene editing *in vivo*.

Despite incomplete excision of the HIV-1 viral genome, treatment with CRISPR/Cas9 has profound clinical relevance, as the knockdown of HIV-1 mRNA enhances temporal processing for a protracted period of time. PPI of the ASR, a translational experimental paradigm introduced and popularized by Hoffman and Ison (Hoffman & Searle, 1965; Ison & Hammond, 1971), is utilized to tap temporal processing, a construct analogous to the speed of information processing in humans. Indeed, HIV-1 seropositive individuals with HAND exhibited profound impairments in PPI, assessed using an eyeblink startle experimental paradigm, relative to their cognitively intact counterparts (Minassian et al., 2013). The HIV-1 Tg rat recapitulates and extends the PPI deficits reported in HIV-1 seropositive individuals with HAND, as temporal processing alterations in the HIV-1 Tg rat occur early in development (McLaurin et al., 2017b), progress across time (Moran et al., 2013; McLaurin et al., 2016; McLaurin et al., 2018b), and mediates alterations in higher-order cognitive processes (McLaurin et al., 2019a). The ability of PPI to partially mediate the relationship between HIV-1 and higher-order cognitive processes (i.e., learning, sustained attention, and long-term episodic memory; McLaurin et al., 2019a) is of fundamental importance, as these analyses suggest that the enhancement of PPI by CRISPR/Cas9 may lead to the mitigation of HIV-1-associated neurocognitive impairments more broadly. Additional studies remain necessary to optimize the CRISPR/Cas9 treatment conditions and evaluate its therapeutic potential to mitigate alterations in higher-order cognitive processes induced by the constitutive expression of HIV-1 viral proteins.

Taken together, proof-of-concept studies demonstrate the susceptibility of mixed glia to efficacious gene editing via CRISPR/Cas9 *in vitro* and *in vivo*. Fundamental challenges for the complete eradication of the HIV-1 viral genome remain, however, as incomplete excision and significant individual variability were observed. Nevertheless, the clinical relevance of CRISPR/Cas9 cannot be understated, as the knockdown of HIV-1 mRNA partially restores the developmental trajectory of temporal processing. Thus, even in the absence of full viral eradication, gene editing via CRISPR/Cas9 may afford a novel therapeutic strategy for HAND.
